# Repressor of temperate mycobacteriophage L1 harbors a stable C-terminal domain and binds to different asymmetric operator DNAs with variable affinity

**DOI:** 10.1186/1743-422X-4-64

**Published:** 2007-06-28

**Authors:** Tridib Ganguly, Amitava Bandhu, Partho Chattoraj, Palas K Chanda, Malabika Das, Nitai C Mandal, Subrata Sau

**Affiliations:** 1 Department of Biochemistry, Bose Institute, P1/12 CIT Scheme VII M, Kolkata – 700 054, West Bengal, India

## Abstract

**Background:**

Lysogenic mode of life cycle of a temperate bacteriophage is generally maintained by a protein called 'repressor'. Repressor proteins of temperate lambdoid phages bind to a few symmetric operator DNAs in order to regulate their gene expression. In contrast, repressor molecules of temperate mycobacteriophages and some other phages bind to multiple asymmetric operator DNAs. Very little is known at present about the structure-function relationship of any mycobacteriophage repressor.

**Results:**

Using highly purified repressor (CI) of temperate mycobacteriophage L1, we have demonstrated here that L1 CI harbors an N-terminal domain (NTD) and a C-terminal domain (CTD) which are separated by a small hinge region. Interestingly, CTD is more compact than NTD at 25°C. Both CTD and CI contain significant amount of α-helix at 30°C but unfold partly at 42°C. At nearly 200 nM concentration, both proteins form appreciable amount of dimers in solution. Additional studies reveal that CI binds to *O*_64 _and *O*_*L *_types of asymmetric operators of L1 with variable affinity at 25°C. Interestingly, repressor – operator interaction is affected drastically at 42°C. The conformational change of CI is most possibly responsible for its reduced operator binding affinity at 42°C.

**Conclusion:**

Repressors encoded by mycobacteriophages differ significantly from the repressor proteins of λ and related phages at functional level but at structural level they are nearly similar.

## Background

Repressor of a temperate bacteriophage maintains its lysogenic mode of life cycle generally by turning off the transcription of its lytic genes and simultaneously by keeping its own synthesis on. The lysis – lysogeny decisions in lambda and related phages are in fact controlled by binding of two antagonistic transcriptional repressors (e.g. CI and Cro in lambda phage) to two master operators overlapped with divergent early promoters [1]. Nearly similar regulatory circuits controlling the lysogenic – lytic developments have also been detected in phages P2 [2], Mu [3], HK022 [4], Phi 80 [5], and CTXΦ [6]. Lambda repressors bound to *O*_L _and *O*_R _operators octamerize and the resulting DNA loop enhances the repression of early promoters and stably maintains lysogeny [7]. Interestingly, the mechanism of actions of repressors of coliphages P1 and P7 [8,9], mycobacteriophages L5 and Bxb1 [10], and *B. subtilis *phage Phi105 [11] differ considerably from those of lambda and related phages. Repressors of these phages bind to multiple asymmetric operators instead of symmetric operators. Thus far most repressors of the second group phages had not been studied at length.

Mycobacteriophage L1, homoimmune to mycobacteriophage L5, utilizes *M. smegmatis *as its host [10]. Its repressor gene was identified, cloned and characterized to some extent [12-15]. L1 repressor (CI) was found 100% identical to L5 repressor at amino acid sequence level. An L1 promoter [16] that binds to CI specifically was also found 100% identical to L5 early promoter *P*_*left *_[10] at nucleotide level. An asymmetric operator (5'GGTGGCTGTCAAG) that overlaps *P*_*left *_in fact interacts with CI. Interestingly, L5 harbors eleven more operators of the above type at different places of its genome. A second predominant group which consists of seven other identical L5 operators (5'GGTGGATGTCAAG) differs from the former group by a single base [10]. There are eleven other asymmetric operators in L5 and they carry 1 – 2 base changes at different positions except 6^th ^position. Among these 3^rd ^group operators, five operators interact with CI. L5 operators were shown not only to repress the transcription from its early promoters but also to stop the elongation of L5 transcripts. Additional studies reveal that affinity of CI to an L1 operator (5'GGTGGCTGTCAAG) decreases notably at 42°C compared to that at 32°C [14]. A mutant CI devoid of its helix-turn-helix (HTH) DNA binding motif does not bind to operator at 32° – 42°C [15], whereas another mutant CI carrying a point mutation at its C-terminal end, binds to operator at 32°C but not at 42°C. Thus far, little study was carried out to understand the structure of L1/L5 repressor and its molecular mechanism of interaction with asymmetric operator DNA.

Our preliminary studies indicate that an L1 DNA region is 100% identical to an L5 DNA region harboring L5 gp*64 *gene and an upstream operator (5'GGTGGATGTCAAG) [10]. In this communication, we have designated the L1 DNA fragments carrying operators 5'GGTGGATGTCAAG and 5'GGTGGCTGTCAAG as *O*_64 _and *O*_*L*_, respectively, and shown that CI binds to former operator more strongly than latter operator. Interestingly, repressor-operator interaction is drastically affected at 42°C. We also report for the first time that L1 repressor possesses two domains (an N-terminal domain, NTD and a C-terminal domain, CTD) at room temperature. CTD is comparatively more compact than NTD at room temperature. Both CI and CTD carry significant amount of α-helix at 30°C but unfold partly at 42°C. Both proteins also form appreciable amount of dimers in solution.

## Results and Discussion

### L1 repressor possesses two domains

**Figure 1:**
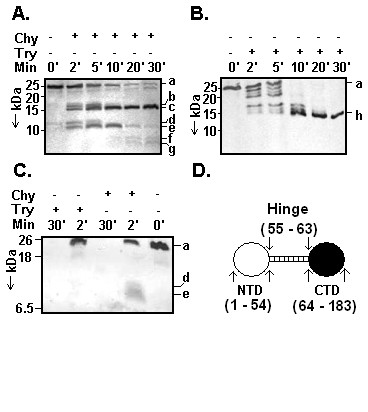
**Domains of L1 repressor.** Chymotrypsin **(A) **and trypsin **(B) **digested His-CI fragments were analyzed by Tris-Tricine SDS-16.5% PAGE followed by silver staining. Molecular masses (in kDa) of marker proteins are shown at the left side of gel. 'Chy' and 'Try' indicate chymotrypsin and trypsin, respectively whereas, a – h indicate intact repressor, different digested fragments of repressor, respectively. N-terminal ends of fragments c and h were sequenced. **(C) **Western blotting analysis of chymotrypsin/trypsin digested His-CI fragments from 2 and 30 mins incubations by a standard procedure as indicated in Materials and method. **(D) **Summary of proteolysis. The putative domains of CI and its amino acid residues involved in formation of hinge, NTD and CTD are indicated.

Many repressors possess domains, which perform distinct function [1,6,9,19,20]. To detect domain (s) if present any in CI, limited proteolysis of His-CI was performed with chymotrypsin and trypsin separately according to standard techniques. As shown in Fig. 1A, two major protein fragments of nearly 16 and 10.5 kDa (designated c and e, respectively) were generated from intact repressor (designated a) upon digestion with chymotrypsin for 2 mins at 25°C. While fragment c remained undigested over the entire period of digestion, fragment e was degraded gradually followed by the appearance of some smaller fragments designated f and g. Some less prominent fragments such as fragments b and d were not seen after 5 min. Surprisingly, the fragment c was not digested further even for ~12 h incubation with chymotrypsin though its cleavage sites are distributed all over CI (not shown). Further analysis showed that only fragments d and e of 2 mins digestion products reacted with anti-his antibody along with the intact repressor (Fig. 1C). No other fragments derived from 2 and 30 mins digestions interacted with anti-his antibody. Sequence of the first ten N-terminal amino acid residues of fragment c was determined to be GGRLTTRQIV. These 1–10 amino acid residues were found 100% identical to the 92 – 101 (equivalent to 56 – 65 amino acid residues of CI) amino acid residues of His-CI. As the size of fragment c appeared unchanged over the whole digestion period and fragment d and e disappeared with increasing time of digestion, fragments f and g might have originated from the internal regions of fragment d and/or e.

Contrary to chymotrypsin digestion, approximately six protein fragments having molecular weights in the range of ~23 to ~15 kDa were generated with trypsin at early period of digestion (Fig. 1B). All these fragments except one (designated h) disappeared gradually with the longer time of digestion. Intensity of fragment h having molecular mass of ~15 kDa increased with time and reached to maximum at 30 min. Analysis of 2 and 30 mins digestion samples revealed that none of the fragments reacted with anti-His antibody whereas the full-length did (Fig. 1C). The first ten N-terminal amino acid residues of fragment h were found to be QIVQQNWPWD. These amino acid residues in fact correspond to 99 – 108 amino acid residues of His-CI (equivalent to 63 – 72 amino acid residues of CI). Taking together, the data indicate that CI indeed possesses domain structure and the most flexible or exposed region of CI is located around its 55 – 63 amino acid residues. The putative exposed region is designated as 'hinge' region here. The hinge region of CI is thus flanked by an NTD and a CTD that encompass through ~1 – 54 and ~64 – 183 amino acid residues, respectively (Fig. 1D). As the putative HTH motif is located within 34 – 53 amino acid residues of CI [13,15], hinge region may not be extended much in its left ward direction but may be extended up to the 106^th ^residue at right ward direction. The 107^th ^residue, a tryptophan is buried in His-CI (equivalent to 70^th ^tryptophan residue in CI) as evident from analysis of chymotrypsin digested fragments of His-CI. It is interesting to note that size of putative hinge region in L1 CI is less than half of that of λ phage [1]. The data also indicate that CTD is comparatively more compact than NTD at room temperature.

### CD spectra of CI, His-CI and CTD

**Figure 2:**
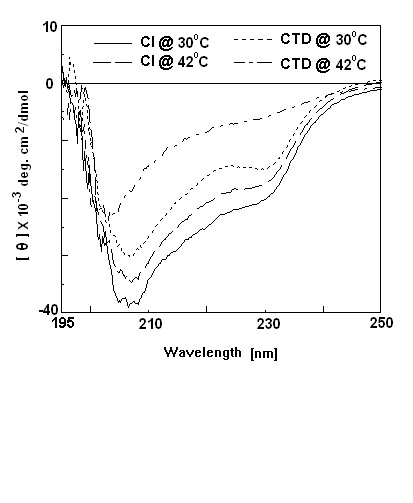
**CD-spectra of His-CI and CTD**. Far UV CD-spectra of His-CI and CTD (64–183 amino acid residues) were measured at 30° and 42°C separately in Buffer A [50 mM phosphate buffer (pH-6.0), 50 mM NaCl, 1 mM EDTA, 5% glycerol].

CD spectra measurement of proteins can predict about their secondary structural elements and conformational changes under different environmental conditions. To get clues about the secondary structures in CI, His-CI, and CTD and also to see the effect of temperature on their conformations, their CD spectra (200–260 nm) were recorded at different temperatures. As shown in Fig. 2, the spectrum of His-CI obtained at 30°C shows a peak of large negative ellipticity at ~208 nm. This indicates that there is a substantial amount of α-helical structure in His-CI at 30°C. Analysis of the spectrum by a software program CDNN [21] in fact showed that there were about 22.2% α-helix, 23.3% β strand, and 37.2% coil in His-CI. Native CI also shows nearly identical CD spectrum at 30°C and was found to carry ~29.9% α-helix, 20% β strand, and 34.2% coil (data not shown). The peak of the CD spectrum of His-CI was however reduced substantially at 42°C (Fig. 2). There were nearly 23% reduction of α-helical structure and concomitant ~10% increase of random coil in His-CI when temperature was increased from 30° to 42°C. The CD spectrum of CTD recorded at 30°C also showed a peak of negative ellipticity near 208 nm which was reduced substantially when the incubation temperature was raised to 42°C. This temperature increase was found to reduce the α-helical content in CTD by nearly 34%, whereas random coil increased to about 26% under identical condition. The data together indicate that there are considerable amount of unfolding as well as conformational change of each of His-CI and CTD at 42°C compared to those at 30°C.

### Both CI and CTD dimerize in solution

**Figure 3:**
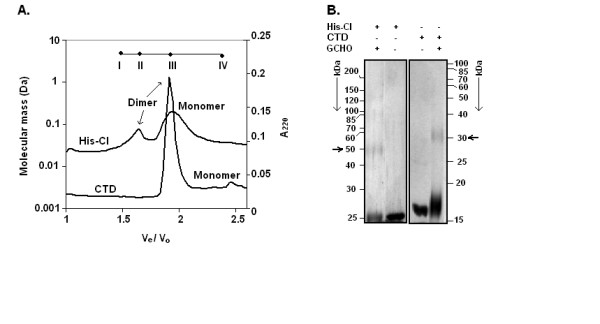
**Oligomerization of His-CI and CTD**. **(A) **Gel filtration analysis. Each protein was loaded onto HPLC gel filtration column and absorbance of eluted fractions was determined at 220 nm. Column was calibrated with BSA (66 kDa, I), ovalbumin (46 kDa, II), carbonic anhydrase (29 kDa, III), and lysozyme (14.4 kDa, IV). Molecular weights were plotted against *V*_*e*_/*V*_*o*_, where *V*_*e *_and *V*_*o *_denote elution volume and void volume respectively. Void volume of column was determined from elution of blue dextran. **(B) **Glutaraldehyde (GCHO) cross-linking. Nearly 0.5 μM His-CI or CTD was cross-linked with 0.1% GCHO and samples were analyzed by SDS-10% PAGE. Protein bands were visualized by silver staining. Horizontal arrows denote dimeric His-CI and CTD species.

To reveal the oligomeric status of CI and CTD in solution, both gel filtration chromatography and glutaraldehyde crosslinking were carried out according to the standard methods [18]. As shown in Fig. 3A, passage of ~20 μM His-CI through gel filtration column produced two peaks. In comparison with the elution profiles of some standard proteins (also shown in Fig. 3A), the peaks seemed to be consistent with monomeric (~25 kDa)- and dimeric (~50 kDa) forms of His-CI, respectively. Gel filtration chromatography of ~20 μM CTD also produced two peaks which correspond to dimeric (~28 kDa) and monomeric (~14 kDa) CTD, respectively (Fig. 3A). The dimeric His-CI/CTD species, however, was clearly seen when glutaraldehyde-treated His-CI/CTD solution (500 nM) was analyzed by SDS-PAGE (Fig. 3B). Taken together, the data indicate that both CI and CTD form dimers in solution at hundred nanomolar concentration.

It is interesting to note from the gel filtration analyses that monomeric His-CI and dimeric CTD are the predominant species in solution. The exact reason of the increased amount of monomeric His-CI or the reason of formation of the elevated level of dimeric CTD in solution is not very clear at this moment. It is possible that a part of dimeric His-CI had been destroyed during its run through gel filtration column and removal of N-terminal region of His-CI augments the dimerization of CTD by bringing out some conformational change in latter.

### CI binds more strongly to O_64 _operator than O_L _operator

**Figure 4:**
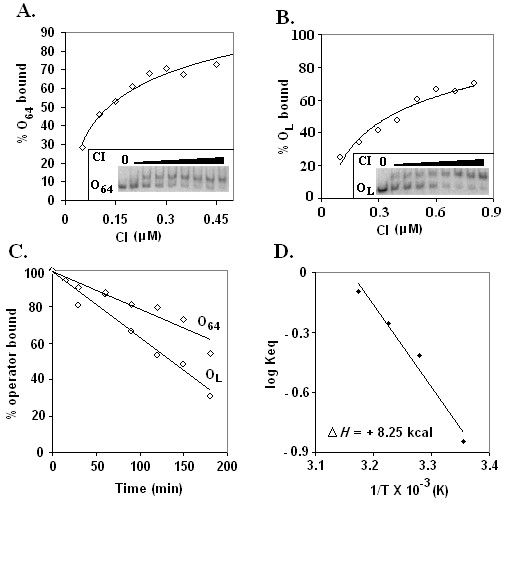
**Equilibrium binding and kinetic study**. Equilibrium binding of CI to *O*_64 _**(A) **and *O*_L _**(B) **operators was studied according to standard method as described in Materials and methods. Plots of % operator bound (estimated from inset gel shift assay pictures) versus CI concentration (0.05 – 0.45 μM and 0.1 – 0.8 μM CI with *O*_64 _and *O*_L_, respectively) are shown. Nearly 0.1 nM labeled operator was used in each reaction. **(C) **Plot of %operator bound versus time shows the kinetics of CI dissociation from *O*_64 _and *O*_L _operators in presences of excess cold operator. The amount of operator bound in the shifted complex of the zero time aliquot was considered as 100%. **(D) **Plot of log Keq versus 1/T shows equilibrium binding of CI to *O*_64 _operator at temperatures ranging from 25° – 42°C. All curves/lines are best-fit curves/lines.

The *O*_64 _and *O*_*L *_types of operators are predominant in L5 [10] and also possibly in L1. To understand the relative affinity of CI to such operators, equilibrium binding of CI as well as dissociation kinetics of repressor-operator complexes was studied by separate gel shift assays. Figs. 4A and 4B show the gel pictures as well as the corresponding plots of equilibrium binding of CI to *O*_64 _and *O*_L_, respectively. At CI concentration that produces 50% saturation of input *O*_64 _operator, the apparent equilibrium dissociation constant is nearly 140 nM. In contrast, apparent equilibrium dissociation constants for CI – *O*_L _interaction is ~370 nM. The data suggest that affinity of CI to *O*_64 _is nearly 2.5 fold higher than that of CI to *O*_*L*_.

Fig. 4C shows the kinetics of dissociation of *O*_64 _– and *O*_*L *_– CI complexes. Both the dissociation reactions appeared to be the first order in nature. While half-life and dissociation rate constant for dissociation of CI-*O*_64 _complex were ~233 min and 2.97 × 10^-3 ^s^-1^, respectively, and for CI-*O*_*L *_complex were 135 min and 5.13 × 10^-3 ^s^-1^, respectively. The data support the suggestions made from the equilibrium binding study.

Temperatures greater than 32°C were shown to affect CI – operator interaction severely [14]. Equilibrium binding study in fact showed that there was about 6 fold decrease of CI affinity to *O*_64 _when temperature increased from 25° to 42°C. This most possibly happens due to the conformational change of CI at 42°C (as evident from CD spectra measurement, see above). It was also found that Van't Hoff plot is linear for 25° – 42°C (Fig. 4D) and the associated enthalpy change of operator binding is nearly 8 kcal/mol. This enthalpy change is possibly involved with the binding of CI to *O*_64 _operator DNA.

The stronger affinity of repressor to *O*_64 _operator may be due to the fact that 6^th ^position base 'A' of *O*_64 _contributes more to CI binding than base 'C' of *O*_L _operator located at identical position. Additional equilibrium binding studies in fact revealed that affinity of CI to a 21 bp DNA fragment (carrying 5'GGTGGATGTCAAG) is about 2.57 fold higher than that to another 21 bp DNA fragment harboring 5'GGTGGCTGTCAAG (data not shown). The result is however unusual as *O*_L _operators are located mostly at the ends of L5 genome (especially, in R*cos *end) including one that overlaps with the early promoter of L5/L1 [10,15]. In contrast, most *O*_64 _operator sites are distributed in ~4 – 41 Kbp region of L5 genome that encodes putative phage-specific head, tail, DNA replication proteins etc. [10]. Such organization of *O*_64 _like operators over the L1/L5 genome suggests that they are possibly utilized to ensure the complete repression of expression of L1/L5 late and delayed early genes during lysogenic development. This type of unexpected mechanism of gene expression in mycobacteriophages L1 and L5 is partly supported by the fact that only a few repressor-regulated promoters [10,16] have been cloned from L1 and L5 phages so far.

It was noticed by us and also by Hatfull's group [10] that L1/L5 repressor binds to its cognate operator at nearly hundred-nanomolar concentration. At concentration close to 200 nM (i.e. apparent equilibrium dissociation constant for CI – operator interaction, Figs. 4A and 4B), the CTD (64–183) predominantly forms dimer in solution, whereas, L1 repressor exists as a mixture of monomer and dimer (Fig. 3). If CI binds to operator as dimer, then a huge amount of repressor would be required to bind to all the 30 operators [10]. The repressor concentration in lysogen was not determined so far in L1/L5 lysogen. The Hill plots calculated from our equilibrium binding data (Figs. 4A and 4B) of repressor yielded best fit straight lines with slopes very close to 1 (data not shown) and addition of operator DNA did not increase the amount of dimeric repressor in solution (our unpublished data). Taking together we speculate that L1 repressor possibly binds to cognate operator as monomer. The role of dimeric repressor in L1/L5 phage development is not known with certainty at present.

## Conclusion

Research on repressor proteins of temperate mycobacteriophages L1, L5, and BxB1 during the last twelve years showed that they regulate gene expression by a mechanism different from those of well-studied λ and related phage repressors. Our data here indicate that the basic structures of repressor proteins of mycobacteriophages are quite similar to those of later phages.

## Methods

### Bacterial and phage strains, plasmids and growth conditions

*M. smegmatis *mc^2^155 and *E. coli *were routinely grown in Middlebrook 7H9 [12] and Luria-Bertani [17] media (supplemented with appropriate antibiotics), respectively. The vectors pSD5S30 and pMPMK4 were obtained from Drs. A. Tyagi (University of Delhi, India) and S. Yasuda (Japan), respectively. Mycobacteriophage L1 and its growth conditions were described previously [12].

### Purification of L1 repressor

To purify CI, cells harvested from one liter induced E. coli (pSAU1049) culture [14] were resuspended in 1/20 volume of lysis buffer A [20 mM Na-phosphate buffer (pH 6.0), 50 mM NaCl, 1 mM EDTA, 5% glycerol, and 100 μg/ml PMSF] followed by preparation of crude extract by appropriate sonication. Crude extract without cell debris was subjected sequentially to ultracentrifugation, 40 – 65% ammonium sulfate precipitation, SP-Sepharose column chromatography and hydroxyapatite column chromatography and fractions collected from each step were analyzed by SDS-12%PAGE (Fig. S1A). The elute from final step mainly shows a protein of ~22 kDa protein. It might be L1 CI as its molecular weight closely matched to that estimated from amino acid sequence of CI and binds to L1 operator DNA (Fig. S1C). The putative repressor was estimated to be around 97% pure.

To overexpress CI as an N-terminal histidine-tagged variant (His-CI), a vector pSAU1180 was constructed by cloning an L1 DNA [12,17] (amplified with primers, LCP2: 5'AAGCTTCCTTTCGTTGCGCGGC and LCP3: 5'GAATTCATGAGCGGCAAAATC) to pET28a (Novagen, USA). This cloning has added extra 36 amino acid residues (including six histidine residues) to N-terminal end of CI.

Histidine-tagged CI (His-CI) overexpressed in *E. coli *BL21 (DE3) (pSAU1180) cells was purified by Ni-NTA resin (QIAGEN, Germany) according to manufacturer's protocol. Analysis of elution fraction showed only one protein of nearly 25 kDa (Fig. S1B). This seems to be the His-CI as its molecular mass matched to that estimated from its primary structure and it binds to L1 operator DNA (Fig. S1C).

### Limited proteolysis of His-CI

It was carried out at 25°C in 20 μl phosphate buffer [50 mM phosphate buffer (pH 6.0), 50 mM NaCl]. Nearly 4 μg His-CI was mixed with 16 ng enzyme and reactions were performed for different times ranging from 0 – 30 mins followed by analysis of samples by Tris-Tricine SDS-16.5% PAGE [18].

### Western Blotting

Protein fragments generated from limited proteolysis of His-CI were transferred to nitrocellulose membrane followed by treatment of membrane sequentially with 3% BSA, mouse anti-his antibody (QIAGEN, Germany), goat anti-mouse antibody IgG1-AP (Santa Cruz Biotechnology, Germany), and NBT – BCIP (Bangalore Genei, India) solution for 1–2 h at room temperature. Each incubation step follows adequate washing step.

### N-terminal protein sequencing

Stable His-CI fragments obtained from limited proteolysis were transferred to PVDF membrane. A PVDF paper strip carrying the fragment of interest was utilized for its N-terminal sequencing according to a standard protocol (Applied Biosystems, USA).

### Purification of CTD

To purify CTD, nearly 200 μg of His-CI was digested with 800 ng of trypsin in 400 μl for 30 minutes at 25°C. After dialysis against buffer B, digested protein was loaded onto Ni-NTA column followed by the collection of flow-through. Analysis shows that flow-through contains mainly CTD (data not shown).

### Cloning of *O*_64 _and *O*_*L *_operators

The 40426 – 40812 bp co-ordinate of L5 genome carries gp*64 *gene and an operator (5'GGTGGATGTCAAG) [10]. A 386 bp DNA was amplified from L1 genomic DNA using primers designed on the basis of L5 *gp64 *and neighboring sequences and analysis revealed that it is 100% identical to the above mentioned L5 region at nucleotide level (data not shown). Next, polymerase chain reaction was carried out using 386 bp L1 DNA as a template and a suitable primer pair and the resulting ~120 bp DNA fragment harboring 5'GGTGGATGTCAAG sequence was designated *O*_64_.

Cloning of a 97 bp L1 DNA fragment that harbors a promoter and an operator (5'GGTGGCTGTCAAG) was reported previously [14] and designated *O*_L _here.

### Gel shift assay

To study the equilibrium binding of CI to *O*_64 _and *O*_L _operators, several gel shift assay were performed according to a modified method described earlier [14]. Briefly, a 20 μl reaction mixture in Buffer A containing repressor, [^32^P-γ] ATP (BARC, India) end labeled operator DNA and 10 μg/ml bovine serum albumin was incubated at 25°C for 20 mins. As a reaction between L1 repressor and cognate operator is very fast [15], we assumed that 20 mins are sufficient for reaching equilibrium between the two species. Analysis of reaction mixtures was performed by a standard method as described earlier [14,18].

Using different temperature-controlled water baths, equilibrium dissociation constant (Keq) of operator – CI interaction at each of 32°, 37°, and 42°C was determined from respective gel shift assay picture data.

To study the rate of dissociation of CI – operator complexes, a 200 μl reaction mixture in Buffer A containing ~0.1 nM operator and saturating amount of repressor was incubated for 20 mins at 25°C. Then a 300-fold excess of cold operator was added to the reaction mixture and 20 μl aliquot taken out at 0, 10, 20, 30, 60, 90, 120, 150 and 180 mins. Analysis of reaction mixture was done by same procedure as described above.

### CD spectra of CI and CTD

Nearly 20 μM protein was taken in a cuvette (1 mm path length) and incubated at 32° or 42°C for 10 min. Next, Circular Dichroism (CD) spectrum (200 – 260 nm) of the protein was recorded by JASCO J600 spectrophotometer.

### Analytical gel filtration chromatography

Analytical gel filtration chromatography was performed in an HPLC system using a gel filtration column Protein Pak (Waters, USA) after equilibration with 1× Buffer A (minus PMSF).

### Glutaraldehyde cross-linking

Cross-linking reactions of His-CI and CTD [56–183] were performed in Buffer A in 20 μl total volume at 25°C. Repressor containing solution was incubated at 25°C for 20 mins. Next, glutaraldehyde solution (0.1%) was added to repressor solution and incubated for 2 mins. The reaction was stopped by adding 5 μl of 4× SDS gel loading dye. After boiling the sample for 2 mins, it was analyzed by 10% SDS PAGE.

## Competing interests

The author(s) declare that they have no competing interests.

## Authors' contributions

TG has performed most of the experiments described here. AB has carried out a part of work of this manuscript. PC, PKC, and MD have contributed significantly in data interpretation, editing and presentation. NCM has provided valuable inputs in modifying experimental design and data interpretation. SS has designed most of the experiments, supervised the work, procured fund for the work, and prepared the manuscript.

## Supplementary Material

Additional File 1 **Supplementary figure S1. Purification and partial characterization of native and N-terminal histidine tagged L1 repressors**. **(A) **SDS – 10% polyacrylamide gel electrophoresis of protein samples collected from different steps of purification of native L1 repressor. Nearly 10 μg protein was loaded in each lane. Lane 1, fraction I (crude extract); 2, fraction II (after ultracentrifugation); 3, fraction III (after 40 – 65% ammonium sulfate precipitation); 4, fraction IV (after ion exchange chromatography by SP-Sepharose HP column), 5, Fraction V (after hydroxyapatite column chromatography). The molecular weight (in kDa) marker was indicated at the left side of gel picture. Arrow indicates purified repressor. **(B) **SDS – 12% PAGE of different protein fractions carrying N-terminal histidine tagged L1 repressor. Each lane carries about 10 μg protein. Lane 1, crude extract from uninduced cells (after removal of cell debris); 2, crude extract from induced cells (after removal of cell debris); lane 3, flow – through fraction; 4, wash fraction; 5 – 6, elution fractions from Ni-NTA column. Arrow indicates purified repressor. **(C) **DNA binding affinity of different L1 repressors to different DNA fragments. Both [^32^P-γ] ATP end labeled non-specific DNA (135 bp *Eco*RV – *Sa*lI fragment carrying truncated *XylE *gene) and L1 phage-specific operator *O*_L _DNA were incubated with indicated amount of repressor for 20 min at room temperature followed by analysis of all samples by native 6% PAGE. See Experimental for details. Click here for file
