# Supplementary figures and images for: Repressor of temperate mycobacteriophage L1 harbors a stable C-terminal domain and binds to different asymmetric operator DNAs with variable affinity
Source: Virol J. 2007 Jun 28;4:64. doi: 10.1186/1743-422X-4-64 (PMC1934351; doi:10.1186/1743-422X-4-64)

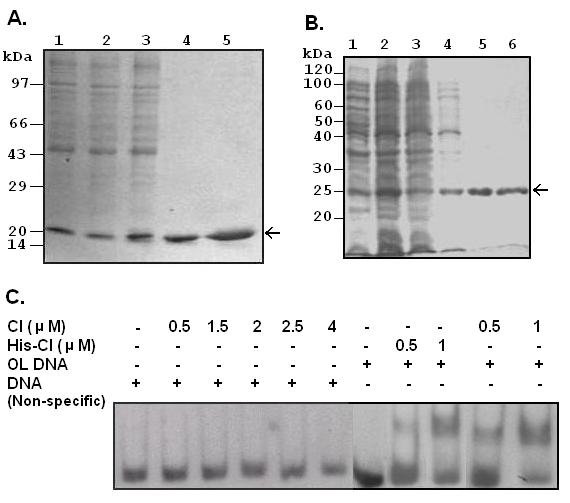

Supplement: Additional File 1 — Supplementary figure S1. Purification and partial characterization of native and N-terminal histidine tagged L1 repressors. (A) SDS – 10% polyacrylamide gel electrophoresis of protein samples collected from different steps of purification of native L1 repressor. Nearly 10 μg protein was loaded in each lane. Lane 1, fraction I (crude extract); 2, fraction II (after ultracentrifugation); 3, fraction III (after 40 – 65% ammonium sulfate precipitation); 4, fraction IV (after ion exchange chromatography by SP-Sepharose HP column), 5, Fraction V (after hydroxyapatite column chromatography). The molecular weight (in kDa) marker was indicated at the left side of gel picture. Arrow indicates purified repressor. (B) SDS – 12% PAGE of different protein fractions carrying N-terminal histidine tagged L1 repressor. Each lane carries about 10 μg protein. Lane 1, crude extract from uninduced cells (after removal of cell debris); 2, crude extract from induced cells (after removal of cell debris); lane 3, flow – through fraction; 4, wash fraction; 5 – 6, elution fractions from Ni-NTA column. Arrow indicates purified repressor. (C) DNA binding affinity of different L1 repressors to different DNA fragments. Both [32P-γ] ATP end labeled non-specific DNA (135 bp EcoRV – SalI fragment carrying truncated XylE gene) and L1 phage-specific operator OL DNA were incubated with indicated amount of repressor for 20 min at room temperature followed by analysis of all samples by native 6% PAGE. See Experimental for details. [file 1743-422X-4-64-S1.DOC]
